# Engineering the cell surface display of cohesins for assembly of cellulosome-inspired enzyme complexes on *Lactococcus lactis*

**DOI:** 10.1186/1475-2859-9-69

**Published:** 2010-09-14

**Authors:** Andrew S Wieczorek, Vincent JJ Martin

**Affiliations:** 1 Department of Biology, Concordia University, Montréal, Québec, H4B 1R6, Canada

## Abstract

**Background:**

The assembly and spatial organization of enzymes in naturally occurring multi-protein complexes is of paramount importance for the efficient degradation of complex polymers and biosynthesis of valuable products. The degradation of cellulose into fermentable sugars by *Clostridium thermocellum *is achieved by means of a multi-protein "cellulosome" complex. Assembled via dockerin-cohesin interactions, the cellulosome is associated with the cell surface during cellulose hydrolysis, forming ternary cellulose-enzyme-microbe complexes for enhanced activity and synergy. The assembly of recombinant cell surface displayed cellulosome-inspired complexes in surrogate microbes is highly desirable. The model organism *Lactococcus lactis *is of particular interest as it has been metabolically engineered to produce a variety of commodity chemicals including lactic acid and bioactive compounds, and can efficiently secrete an array of recombinant proteins and enzymes of varying sizes.

**Results:**

Fragments of the scaffoldin protein CipA were functionally displayed on the cell surface of *Lactococcus lactis*. Scaffolds were engineered to contain a single cohesin module, two cohesin modules, one cohesin and a cellulose-binding module, or only a cellulose-binding module. Cell toxicity from over-expression of the proteins was circumvented by use of the *nisA *inducible promoter, and incorporation of the C-terminal anchor motif of the streptococcal M6 protein resulted in the successful surface-display of the scaffolds. The facilitated detection of successfully secreted scaffolds was achieved by fusion with the export-specific reporter staphylococcal nuclease (NucA). Scaffolds retained their ability to associate *in vivo *with an engineered hybrid reporter enzyme, *E*. *coli *β-glucuronidase fused to the type 1 dockerin motif of the cellulosomal enzyme CelS. Surface-anchored complexes exhibited dual enzyme activities (nuclease and β-glucuronidase), and were displayed with efficiencies approaching 10^4 ^complexes/cell.

**Conclusions:**

We report the successful display of cellulosome-inspired recombinant complexes on the surface of *Lactococcus lactis*. Significant differences in display efficiency among constructs were observed and attributed to their structural characteristics including protein conformation and solubility, scaffold size, and the inclusion and exclusion of non-cohesin modules. The surface-display of functional scaffold proteins described here represents a key step in the development of recombinant microorganisms capable of carrying out a variety of metabolic processes including the direct conversion of cellulosic substrates into fuels and chemicals.

## Background

Macromolecular enzyme complexes catalyze an array of biochemical and metabolic processes such as the degradation of proteins [1,2] or recalcitrant polymers [3] as well as the high-yield synthesis of valuable metabolic products via substrate channeling [4]. From a biotechnological perspective, mimicking such process by incorporating catalytic modules or enzymes of interest within synthetic complexes can significantly enhance the efficiency of such bioprocesses via substrate channeling [5] and increased enzyme synergy [3]. In a cellulosome, multiple enzymes assemble into a macromolecular complex by their association with a scaffold protein for the efficient degradation of cellulose [6]. In the case of the gram-positive thermophile *Clostridium thermocellum*, the cellulosome is anchored to the surface of cells, resulting in one of the most efficient systems for bacterial cellulose hydrolysis [3,7].

Cellulosomal enzymes bear C-terminal type 1 dockerin (dock1) modules, which target them to a central scaffold protein (CipA) via chemically and thermally stable non-covalent interactions with one of nine cohesin (coh) modules [8]. CipA also contains a type 3a cellulose-binding module (CBM3a), allowing the different cellulases to act in synergy on the crystalline substrate, as well as a type 2 dockerin module which binds anchor proteins, ensuring the cellulosome's attachment to the cell [9,10]. Therefore, the architecture of the cellulosome establishes proximal and synergistic effects of enzymes within the complex when associated with the substrate [8,11,12]. These synergistic effects are further augmented by an extra level of synergy resulting from the cellulosome's association with the surface of cells, yielding cellulose-enzyme-microbe (CEM) ternary complexes [6,7,13-18]. CEM ternary complexes benefit from the effects of microbe-enzyme synergy, ultimately limiting the escape of hydrolysis products and enzymes, increasing access to substrate hydrolysis products, minimizing the distance products must diffuse before cellular uptake occurs, concentrating enzymes at the substrate surface, protecting hydrolytic enzymes from proteases and thermal degradation, as well as optimizing the chemical environment at the substrate-microbe interface [6,7,13-16].

In this work, we describe the cell surface display of small cellulosome scaffold proteins in *Lactococcus lactis*, a first and necessary step for the eventual engineering of extracellular protein complexes in this, and other bacterial hosts. "Mini" scaffold proteins have been intracellularly expressed and purified from hosts such as *Escherichia coli *or *Bacillus subtilis *for the purpose of assembling mini-cellulosomes *in vitro *[19-23]. The production of mini-cellulosomes *in vivo *has also been reported in *Clostridium acetobutylicum *and *B. subtilis*, however, complex localization was limited to secretion into the culture supernatant [24,25]. More recently, the surface-display of mini-cellulosomes was described in *Saccharomyces cerevisiae*, in some cases enabling growth on cellulosic substrates [26-29]. However, there have been no reports on the recombinant assembly of cellulosome-inspired complexes on the surface of bacterial cells. For this purpose, we chose *L. lactis*, a gram-positive bacterium with established commercial value. *L. lactis *is of specific interest as it is generally regarded as safe (GRAS), has been used to produce valuable commodity chemicals such as lactic acid [30] and bioactive compounds [31], and has been successfully engineered to secrete and/or display on its cell surface, a wide variety of proteins ranging from 9.8 to 165 kDa [32]. The metabolic engineering tools available in conjunction with the successful controlled expression and high-yield production of enzymes and proteins [32] make it an ideal candidate for the recombinant expression of cellulosomal components. Using *L. lactis *as a surrogate host, we successfully secreted fragments of CipA (CipA_frags_) to the cell surface and the scaffolds retained functionality. All scaffolds containing functional cohesins were capable of associating with an engineered test enzyme, *E. coli *β-glucuronidase (UidA) fused with a dockerin. We envision expanding on this work to eventually engineer larger scaffolds that will serve as the basis for assembling and immobilizing large extracellular enzyme complexes.

## Results

### Regulated expression of CipA_frags _yields the surface-display of scaffold proteins

**Figure 1:**
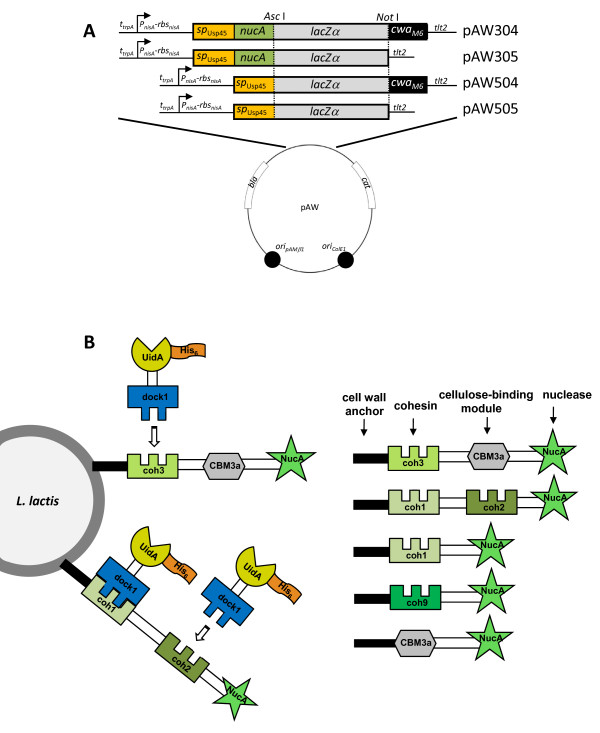
**pAW series of *cipA***_***frag***_**expression vectors and strategy for complex assembly**. **(A) **Vectors were designed for facilitated insertion of fragments of the gene encoding the cellulosomal scaffold protein CipA, into *Asc*I-*Not*I restriction sites. Scaffolds can be optionally expressed with or without an N-terminal nuclease reporter and/or a C-terminal cell wall anchor motif. pAW304 is designed for expression, secretion, and cell wall-targeting of CipA fragments (CipA_frags_) as fusions with the N-terminal NucA reporter. pAW305 is designed for the expression and secretion of CipA_frags _as a fusion with the N-terminal NucA reporter, but without the C-terminal anchor motif. pAW504 is designed for expression, secretion, and cell wall-targeting of CipA_frags _without the N-terminal NucA reporter. pAW505 is designed for the expression and secretion of CipA_frags _with neither the N-terminal NucA reporter nor the C-terminal anchor motif. **(B) **Graphic depiction of the surface-display strategy of engineered scaffolds and their association with the β-glucuronidase-dockerin fusion protein (UidA-dock1). All successfully displayed CipA_frags _are portrayed as fusions with both NucA and a cell wall anchor, however were also expressed and tested without these two components.

**Figure 2:**
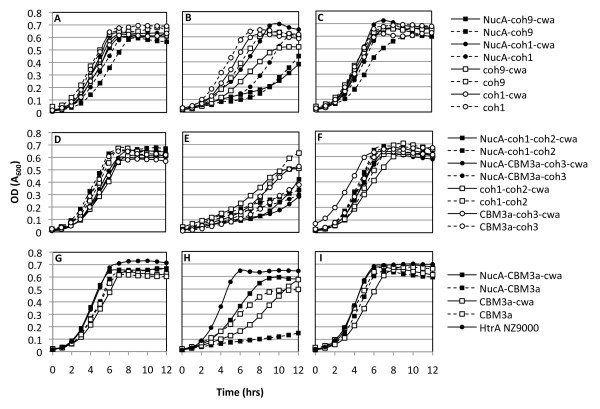
**Growth profiles of *L. lactis *expressing CipA_frags _alone or as fusions with M6_cwa _and/or NucA**. Panels A, D and G represent cultures not induced with nisin, panels B, E, H represent cultures induced with 10 ng/mL nisin at inoculation (t = 0 hrs), and panels C, F, I represent cultures induced with 10 ng/mL nisin in log phase corresponding to an OD_600 _≈ 0.3 (t = 4 hrs). Constructs were grouped according to their modular nature. Top panels depict constructs containing a single cohesin; Middle panels depict constructs containing two CipA modules; Lower panels depict constructs containing no cohesin modules. Black shapes indicate scaffolds containing a fusion with NucA, and white shapes indicate scaffolds where NucA has been removed. Solid lines represent scaffolds expressed with a cell wall anchor, and dotted lines represent scaffolds lacking the cell wall anchor. Experiments were repeated three times yielding identical trends between growth profiles.

*L. lactis *HtrA NZ9000 cells were successfully transformed with either the pAW500 series or pAW300 series of vectors (Fig. 1A), resulting in strains expressing fragments of CipA (CipA_frags_) alone, or as fusions with the NucA export-specific reporter, and/or the cwa_M6 _for anchoring of the scaffold to the cell-surface (Fig. 1B). Growth curves of engineered *L. lactis *strains were used to determine if the expression and secretion of scaffold proteins resulted in growth inhibition. Results from the growth experiments showed a correlation between *cipA*_frag _gene expression and growth inhibition (Fig. 2). The constitutive over-production of recombinant proteins targeted to the cell surface in *L. lactis *may interfere with the integrity of the cell wall [33], whereas in *C. thermocellum*, the constitutive expression of CipA is modulated through catabolite repression [34]. In the absence of the inducer nisin, all *cipA*_frag_-expressing strains grew similarly to the control *L. lactis *HtrA NZ9000 with a final cell density corresponding to an OD_600 _approaching 0.7 (Fig. 2A, D, G). This indicated that little change in growth profile resulted from any leaky expression of the recombinant proteins. Nisin induction at inoculation resulted in cellular toxicity, as demonstrated by extended lag phases, lower growth rates and final cell yields (Fig. 2B, E, H). In all cases, when induction of protein expression was carried out after 4 hrs of growth (corresponding to an OD_600 _≈ 0.3), cultures did not display growth retardation and final cell densities were similar to those attained with no induction (Fig. 2C, F, I). Expression of the various *cipA*_frags _from the constitutive *P*_*59 *_promoter consistently resulted in plasmid rearrangements as observed by restriction digest analysis of the rescued plasmids from both *E. coli *and *L. lactis *(data not shown). From these results, we hypothesized that unregulated high-level expression of the CipA_frag _proteins was toxic to the cells and using a constitutive promoter such as *P*_*59 *_induced plasmid rearrangements that abolished or reduced *cipA*_frag _expression. These results confirmed the necessity for regulating expression of the proteins, which was achieved using the *P*_*nisA *_promoter. With the exception of cell wall anchored scaffold containing only a cellulose-binding module (CBM3a-cwa) (Fig. 2H), removal of the NucA lowered or eliminated toxicity to the cells, as observed by improved growth rates and yields.

### NucA-CipA_frag _proteins are localized to the cell wall of *L. lactis*

**Figure 3:**
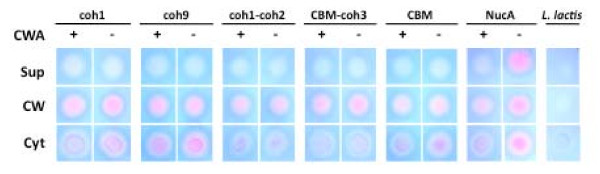
**Cellular localization of NucA-CipA_frag _scaffolds expressed by *L. lactis *with or without M6_cwa_**. NucA activity was detected by spotting cell fractions on TBD-agar and analyzing for pink color formation. Fractions analyzed are supernatant (sup), cell wall (cw), and cytoplasm (cyt). Constructs are represented by their respective CipA_frag _components and were expressed as fusions with NucA with or without cell wall anchor (cwa) domains.

In order to quickly evaluate our success at recombinant protein secretion in *L. lactis*, a nuclease enzyme was fused to the CipA fragments to be displayed on the cell surface. *L. lactis *cells harboring the pAW300 series of vectors all displayed a NucA^+ ^phenotype on plates overlaid with TBD agar, confirming that all variants of the NucA-CipA_frag _proteins were successfully secreted and that the nuclease retained its function when expressed as an N-terminal fusion to CipA_frags_. To determine the cellular localization of the expressed CipA_frag _fusion proteins, cell fractionations were performed, and cytoplasmic, cell wall, and supernatant fractions were spotted on TBD agar. Of the secreted NucA-CipA_frag _proteins, almost all detectable nuclease activity was found in the cell wall fractions corresponding to proteins released from lysozyme/lysostaphin treatments, suggesting successful cell wall targeting of the proteins (Fig. 3). CipA_frag _proteins were not detected in the supernatant, suggesting that secreted proteins remained localized to the cell wall due to the activity of lactococcal sortase. Unexpectedly, the NucA-CipA_frag _fusions lacking the cell wall anchor domain were also detected primarily in the cell wall fractions (Fig. 3) suggesting that fusion of NucA with CipA_frags _caused the scaffolds to remain associated with the cell wall, even without covalent cross-linking by sortase. All of the cytoplasmic fractions were also found to contain varying levels of expressed scaffolds, a finding consistent with observations previously made while exporting recombinant proteins in *L. lactis *[35-38]. We hypothesize that these cytoplasmic proteins were either in the process of being synthesized and exported by the cell via cytoplasmic chaperones, or had evaded the sec-pathway due to a lack of recognition of the signal sequence. In certain instances, the net charge of N-terminal residues downstream of the signal peptide can also contribute to the poor secretion efficiency of recombinant proteins [39]. As expected from previous studies [36,37] in the absence of a cell wall anchor domain, NucA was secreted into the supernatant but remained associated to the cell wall if the anchor domain was present (Fig. 3).

### Cell surface displayed CipA_frag _scaffolds bind UidA-dock1

**Figure 4:**
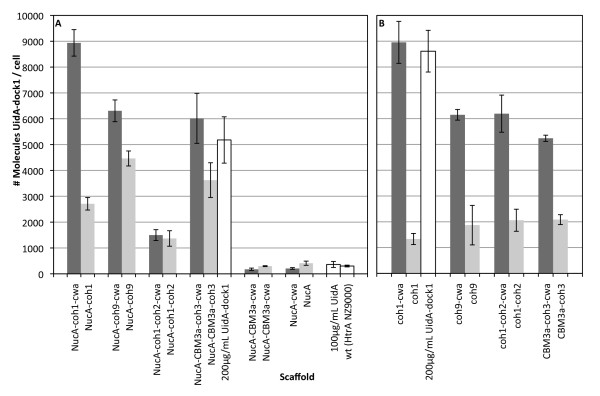
***In vivo *binding of UidAdock1 on live intact *L. lactis *cells displaying CipA_frags_**. CipA_frags _were expressed and anchored as fusions with the NucA reporter enzyme **(A)**, or lacking the NucA reporter **(B)**. Quantification of UidAdock1 molecules bound to *L. lactis *cells corresponds to equivalent amounts of functional cohesin assuming a 1:1 ratio of dockerin-cohesin association. Dark grey bars represent scaffolds containing the C-terminal M6 cell wall anchor motif (cwa), and light grey bars represent their anchor-deficient derivatives. White bars correspond to indicated controls; "200 μg/mL UidA-dock1" represents binding assay carried out with excess enzyme and *L. lactis *pAW328 (NucA-CBM3a-coh3-cwa) to ensure saturation of cohesins. "100 μg/mL UidA" represents binding assay carried out in the presence of UidA and *L. lactis *pAW328 (NucA-CBM3a-cwa). Binding assay carried out with UidA and all other constructs resulted in no association with scaffold-expressing strains (data not shown).

*In vivo *binding assays were performed to determine if a dockerin-containing enzyme could associate with cell surface displayed CipA_frag _scaffold proteins. *L. lactis *cells expressing cell wall and supernatant-targeted scaffolds were incubated with purified β-glucuronidase enzymes fused to a dockerin module (UidA-dock1). After incubation, washed cells were assayed for β-glucuronidase activity, allowing a relative comparison of CipA_frag _display efficiencies between engineered constructs. All constructs containing cohesin modules as part of their scaffolds successfully bound UidA-dock1, while those lacking cohesins as well as the plasmid-free *L. lactis *HtrA NZ9000 failed to do so (Fig. 4). Binding experiments using UidA lacking dock1 resulted in no successful "docking" onto *L. lactis *displaying NucA-CBM3a-coh3 (Fig. 4A) or any other recombinant scaffolds (data not shown). These results demonstrated that functional recombinant scaffolds could be expressed on the surface of *L. lactis *and that cell surface complex formation was dependent on the presence of both cohesin and dockerin modules. Among those strains secreting and displaying functional scaffolds, significant variation in display efficiency was observed. Assuming a 1:1 enzyme-to-cohesin ratio, the approximate number of cohesins and/or scaffolds per cell was determined. The strains that displayed the greatest number of nuclease bearing scaffolds (~9 × 10^3 ^scaffolds/cell) were those expressing the cohesin 1 module alone (coh1-cwa and NucA-coh1-cwa) (Fig. 4). Strains expressing coh9-cwa, NucA-coh9-cwa, coh1-coh2-cwa, CBM3a-coh3-cwa and NucA-CBM3a-coh3-cwa, were estimated to display between 5.0 × 10^3 ^and 6.3 × 10^3 ^scaffolds/cell. These results suggested that the size of the CipA_frag _is not necessarily the limiting factor influencing scaffold display. This was further observed with the relatively lower amount of enzymes binding to *L. lactis *displaying NucA-coh1-coh2-cwa (1.5 × 10^3 ^UidAdock1/cell). Essentially, NucA-coh1-coh2-cwa is of similar size to NucA-CBM3a-coh3-cwa (approx. 68 kDa), contains twice as many cohesins, yet host cells where able to bind one quarter the amount of UidA-dock1 molecules. The predicted molecular weights of the engineered scaffolds were used in order to estimate the net amount of recombinant protein on the cell surface of strains producing scaffolds with a single cohesin. The culture producing the highest net yield of functional recombinant protein was the strain anchoring NucA-CBM3a-coh3-cwa on its surface. Cultures produced and displayed approximately 0.72 mg/mL of recombinant scaffolds, which remained cell-associated and fully functional.

The effect of the N-terminal nuclease reporter on secretion efficiency was also analyzed by comparing the binding capacity of *L. lactis *harboring the pAW300 series (nuclease fusions) with cells harboring the pAW500 (nuclease deficient) series of vectors. Initially included as a reporter to facilitate detection of exported scaffolds, we hypothesized that the nuclease fusion might also increase secretion efficiency, as has been previously observed [35,38]. Removal of NucA had no detrimental effects on scaffold display for all constructs (Fig. 4B), as similar amounts of anchor-containing scaffolds were located to the cell surface. Furthermore, removal of NucA resulted in a fourfold increase in the amount of coh1-coh2-cwa successfully displayed when compared to its NucA-containing counterpart. The presence of NucA appeared to interfere with the secretion of supernatant-targeted scaffolds from the cell, given that the cwa-deficient variants of coh1, coh9, and CBM3a-coh3 remained associated with the cell to a much larger extent than their NucA-deficient counterparts (Fig. 4).

## Discussion

Several recent studies have reported on the recombinant expression of mini cellulosome scaffold proteins in *Saccharomyces cerevisiae *[26-29]. In these examples, the potential application of the engineered strains for the direct conversion of cellulosic biomass to ethanol was the driving factor for choosing *S. cerevisiae *as a host. However, many more platform strains have been or are now being developed that will produce ethanol, biofuels other than ethanol, and non-biofuel chemicals [5,14,40-47]. The economics of these processes would be greatly improved if these engineered microbes could use cellulosic substrates. With this goal in mind, the first logical step in establishing this system was the successful secretion and display of cohesin-bearing scaffold proteins. Previous studies have demonstrated that controlled gene expression in *L. lactis *can reduce toxicity and increase net protein yields [33,48,49]. In our study, the constitutive expression of the scaffold proteins consistently led to cellular toxicity, a problem that was solved by delaying the onset of gene expression until the cells had reached mid log-phase. In cell division, higher concentrations of recombinant cell wall-targeted proteins are localized to the septum, the site of cell wall biosynthesis [33]. It is thus likely that over-expression of our scaffold proteins targeted to the extracytoplasmic space early in the growth phase impaired cell wall biosynthesis and ultimately resulted in cell death. Removal of NucA from the scaffolds decreased or eliminated cellular toxicity for all cohesin-containing constructs (Fig. 2), and we thus suspect that accumulation of NucA in the cytoplasm may also contribute to this observed lag in the onset of growth when induced at t = 0 hrs. In addition, as a larger proportion of scaffolds lacking a cell wall anchor remained trapped in the cell wall when fused with NucA, it is also likely that part of this observed reduction in toxicity is due to a decrease in the amounts of recombinant proteins being trapped in the cell wall and ultimately disrupting its integrity.

Quantification of cell surface displayed proteins in lactic acid bacteria was previously reported using fluorescence-activated cell sorting, flow cytometry, or whole-cell ELISA [50]. In our assay, functionality of the displayed CipA_frag _scaffold proteins could be tested directly through binding with a dockerin-containing reporter enzyme, attesting that the number of cohesins detected was a direct quantification of those that retained biochemical function. Of the four expressed CipA fragments containing at least one cohesin (coh1, coh9, coh1-coh2, CBM3a-coh3), coh1 was displayed with the highest efficiency (~9 × 10^3 ^scaffolds per cell). Due to its small size and decreased number of modules compared with coh1-coh2 and CBM3a-coh3, we attribute part of this increase in display to the decrease in size of the scaffold itself. However, coh1 was also displayed more efficiently than coh9, which is approximately the same size and similar in primary amino acid sequence. One possible explanation may relate to the position of coh1 relative to coh9 on native CipA scaffold. Coh1 is located at the N-terminus of the 200 kDa scaffold CipA, adjacent to the processing site of the signal peptide by the sec-pathway machinery of *C. thermocellum *[7]. It is possible that the increase in secretion efficiency of coh1 when compared with coh9 may be in some part due to differences in amino acid content adjacent to the signal peptide, possibly increasing its accessibility to the chaperones involved in its transport to the extracytoplasmic space [51]. This, however, does not account for the differences in display efficiency between NucA-coh1 and NucA-coh9, as in both cases, NucA is adjacent to the signal sequence. The amount of sequence identity among cohesins perhaps provides a better explanation for these observed differences. Of the nine cohesin modules on CipA, cohesins 3 through 8 show between 96 to 100% sequence identity, whereas among the remaining cohesins, coh1 and coh9 show the least amount of sequence identity (69 and 75%, respectively) [52]. These differences in amino acid content may translate into differences in folding and solubility of the recombinantly expressed modules.

*L. lactis *was engineered to display a scaffold containing 2 cohesin modules (coh1-coh2). Based on a 1:1 binding ratio of the enzyme-cohesin and assuming equivalent expression and secretion, we expected this strain to bind twice the amount of UidA when compared to scaffolds of similar size but containing a single cohesin module (i.e. CBM3a-coh3). However, coh1-coh2 bound similar amounts of UidA as CBM3a-coh3 (Fig 4B). This reduction in UidA binding was not attributed to CipA_frag _size differences, since both mature scaffolds have a theoretical molecular weight of 68 kDa, suggesting that other factors affected secretion and display efficiency. In fact, protein size is not regarded as a major bottleneck for protein secretion in *L. lactis*, as the size of successfully secreted heterologous proteins ranges from 6.9 kDa to a staggering 165 kDa [32]. We hypothesize that the substitution of a cohesin module by CBM3a may have enhanced secretion by increasing the rate of folding of the scaffold into its soluble form. A similar effect was recently reported with the fusion of the highly insoluble *Clostridium cellulovorans *cellulase CelL with the CBM of cellulase CelD, which resulted in dramatic increases in its solubility [53].

Comparisons between amounts of UidA binding to cells expressing CipA_frags _with or without the cwa_M6 _domain revealed that the cell wall anchor motif significantly increased the amounts of functional scaffolds displayed on the cell (Fig. 4). With NucA present, CipA_frags _lacking cwa_M6 _remained cell-associated to a larger extent (Fig. 3) and bound UidA (Fig 4), suggesting that NucA fusion proteins remained trapped in the cell wall for reasons other than covalent cross-linking by the sortase, but yet the cohesin modules were accessible to UidA. This phenomenon is well-documented in other studies of protein secretion in *L. lactis*, as in some cases the fusion of two generally well-secreted proteins results in changes in the folding of the hybrid protein, and deficiencies in their release from cells [37,54]. While the exact mechanism of this phenomenon is not clear, hydrophobic domains resulting from fusing two recombinant proteins may promote cell wall association [37].

## Conclusions

Until now, all attempts to anchor enzymes on the surface of a bacterium have been limited to a single enzyme per anchor [33,35,36,38,50,55-61]. In our system, multiple enzymes could theoretically associate with scaffolds containing a corresponding number of cohesins. We used purified β-glucuronidase fused to a dockerin module as a probe to establish proper display and function of the cohesins, but envision co-expression of enzymes and scaffold in a subsequent development of the strain. We thus envision that further development of this cellulosome-inspired system may contribute to the efficient bioconversion of substrates into industrially relevant fuels and commodity chemicals, and that tailor-designed synthetic macromolecular complexes could be engineered to contain large permutations and combinations of desired enzymes of interest.

## Methods

### Bacterial strains and plasmids

The bacterial strains and plasmids used in this study are listed in Table 1. *E. coli *strains were grown in Luria-Bertani medium at 37°C with shaking (220 rpm). *Lactococcus lactis *HtrA NZ9000 was grown in M17 medium [62] supplemented with 1% (w/v) glucose (GM17) at 30°C without agitation. *C. thermocellum *was grown in ATCC1191 medium at 55°C with 0.2% (w/v) cellobiose as a carbon source. Where appropriate, antibiotics were added as follows: for *E. coli*, ampicillin (100 μg/mL), erythromycin (150 μg/mL), chloramphenicol (10 μg/mL) and kanamycin (30 μg/mL); for *L. lactis*, erythromycin (5 μg/mL) and chloramphenicol (10 μg/mL). General molecular biology techniques for *E. coli *were performed as previously described [63]. Genomic DNA was isolated from *C. thermocellum *as previously described [64]. To make competent cells, *L. lactis *was grown in M17 medium [62] supplemented with 1% (w/v) glucose, 25% (w/v) sucrose and 2% (w/v) glycine and cells were transformed as previously described [65]. M17 media was supplied by Oxoid, LB media was supplied by Novagen , all antibiotics, ρ-nitrophenyl-β-D-glucuronide and nisin were provided by Sigma, and X-gal and IPTG were supplied by Fermentas.

**Table 1:** Strains and plasmids used in this study.

Strain	Genotype/Decription	Source
*L. lactis *HtrA NZ9000	MG1363 (*nisRK *genes on the chromosome)	[37]

*E. coli *TG1	*supE thi-1 *Δ(*lac-proAB*) Δ(*mcrB-hsdSM*)*5 *(rK- mK-) [F' *traD36 proAB lacI*q*Z*Δ*M15*]	ATCC

*E. coli *DH5α	*fhuA2 *Δ*(argF-lacZ)U169 phoA glnV44 Φ80 *Δ*(lacZ)M15 gyrA96 recA1 relA1 endA1 thi-1 hsdR17*	Invitrogen

*E. coli *BL21 (DE3)	*F*^- ^*ompT gal dcm lon hsdS*_*B*_*(r*_*B*_^- ^*m*_*B*_^-^*) λ(DE3 [lacI lacUV5-T7 gene 1 ind1 sam7 nin5])*	Novagen

**Plasmid**		

pVE5524	Ery^r^, Amp^r^; pBS::pIL252::*t*_*trpA*_::*P59*::*rbs*_*usp45*_::*sp*_*Usp45*_*-nucA-cwa*_M6_*-t1t2*	[36]

pVE5523	Ery^r^, Amp^r^; pBS::pIL252::*t*_*trpA*_::*P59*::*rbs*_*usp45*_::*sp*_*Usp45*_*-nucA-t1t2 *	[36]

pSIP502	Ery^r^; *P*_*nisA*__::_*rbs*_*nisA*__::_*uidA*	[66]

pSCNIII	Cm^r^	J. Seegers^*a*^

pUC19	Amp^r^	[69]

pET28(b)	Km^r^	Novagen

pSIPsp-nuc	Ery^r^; *P*_*nisA*_::rbs_nisA_::sp_Usp45_*-nucA*	This Work

pUC104	Amp^r^; *t*_*trpA*_::*P*_*nisA*_::*rbs*_*usp45*_::*sp*_Usp45_*-nucA*	This Work

pUC104mod	Amp^r^; *t*_*trpA*_::*P59*::*rbs*_*usp45*_::s*p*_Usp45_*-nucA*	This Work

pUC304	Amp^r^; *t*_*trpA*_::*P*_*nisA*_::*rbs*_*nisA*_::*sp*_Usp45_*-nucA*	This Work

pUC504	Amp^r^; *t*_*trpA*_::*P*_*nisA*_::*rbs*_*nisA*_::*sp*_Usp45_	This Work

pAW004	Ery^r^, Amp^r^; pBS::pIL252::*t*_*trpA*_::*P59*::*rbs*_*usp45*_::*sp*_Usp45_*-nucA-MCS-cwa*_M6_*-t1t2*	This Work

pAW005	Ery^r^, Amp^r^; pBS::pIL252::*t*_*trpA*_::*P59*::*rbs*_*usp45*_::*sp*_Usp45_*-nucA-MCS-t1t2*	This Work

pAW004Z	Ery^r^, Amp^r^; pBS::pIL252::*t*_*trpA*_::*P59*::*rbs*_*usp45*_::*sp*_Usp45_*-nucA-lacZα-cwa*_M6_*-tlt2*	This Work

pAW005Z	Ery^r^, Amp^r^; pBS::pIL252::*t*_*trpA*_::*P59*::*rbs*_*usp45*_::*sp*_Usp45_*-nucA- lacZα-tlt2*	This Work

pAW004ZC	Cm^r^, Amp^r^; pBS::pIL252::*t*_*trpA*_::*P59*::*rbs*_*usp45*_::*sp*_Usp45_*-nucA-lacZα-cwa*_M6_*-tlt2*	This Work

pAW005ZC	Cm^r^, Amp^r^; pBS::pIL252::*t*_*trpA*_::*P59*::*rbs*_*usp45*_::*sp*_Usp45_*-nucA- lacZα-tlt2*	This Work

pGEMc9	Amp^r^; pGEMT::with cloned *coh9 *from *cipA*	This Work

pGEMc1	Amp^r^; pGEMT::with cloned *coh1 *from *cipA*	This Work

pGEMc1-c2	Amp^r^; pGEMT::with cloned *coh1-coh2 *from *cipA*	This Work

pGEMcbm-c3	Amp^r^; pGEMT::with cloned *cbm3a-coh3 *from *cipA*	This Work

pGEMcbm	Amp^r^; pGEMT::with cloned *cbm3a *from *cipA*	This Work

pAW104	Cm^r^, Amp^r^; pBS::pIL252::*t*_*trpA*_::*P*_*nisA*_::*rbs*_*usp45*_::*sp*_Usp45_*-nucA-LacZα-cwa*_M6_*-tlt2*	This Work

pAW105	Cm^r^, Amp^r^; pBS::pIL252::*t*_*trpA*_::*P*_*nisA*_::*rbs*_*usp45*_::*sp*_Usp45_*-nucA-LacZα-tlt2*	This Work

pAW301	Cm^r^, Amp^r^; pBS::pIL252::*t*_*trpA*_::*P*_*nisA*_::*rbs*_*nisA*_::*sp*_Usp45_*-nucA-cwa*_M6_*-tlt2*	This Work

pAW302	Cm^r^, Amp^r^; pBS::pIL252::*t*_*trpA*_::*P*_*nisA*_::*rbs*_*nisA*_::*sp*_Usp45_*-nucA-tlt2*	This Work

pAW304	Cm^r^, Amp^r^; pBS::pIL252::*t*_*trpA*_::*P*_*nisA*_::*rbs*_*nisA*_::*sp*_Usp45_*-nucA-lacZα-cwa*_M6_*-tlt2*	This Work

pAW305	Cm^r^, Amp^r^; pBS::pIL252::*t*_*trpA*_::*P*_*nisA*_::*rbs*_*nisA*_::*sp*_Usp45_*-nucA-lacZα-tlt2*	This Work

pAW307	Cm^r^, Amp^r^; pBS::pIL252::*t*_*trpA*_::*P*_*nisA*_::*rbs*_*nisA*_::*sp*_Usp45_*-nucA-coh9-cwa*_M6_*-tlt2*	This Work

pAW308	Cm^r^, Amp^r^; pBS::pIL252::*t*_*trpA*_::*P*_*nisA*_::*rbs*_*nisA*_::*sp*_Usp45_*-nucA-coh9-tlt2*	This Work

pAW310	Cm^r^, Amp^r^; pBS::pIL252::*t*_*trpA*_::*P*_*nisA*_::*rbs*_*nisA*_::*sp*_Usp45_*-nucA-coh1-cwa*_M6_*-tlt2*	This Work

pAW311	Cm^r^, Amp^r^; pBS::pIL252::*t*_*trpA*_::*P*_*nisA*_::*rbs*_*nisA*_::*sp*_Usp45_*-nucA-coh1-tlt2*	This Work

pAW334	Cm^r^, Amp^r^; pBS::pIL252::*t*_*trpA*_::*P*_*nisA*_::*rbs*_*nisA*_::*sp*_Usp45_*-nucA-coh1-coh2-cwa*_M6_*-tlt2*	This Work

pAW335	Cm^r^, Amp^r^; pBS::pIL252::*t*_*trpA*_::*P*_*nisA*_::*rbs*_*nisA*_::*sp*_Usp45_*-nucA-coh1-coh2-tlt2*	This Work

pAW328	Cm^r^, Amp^r^; pBS::pIL252::*t*_*trpA*_::*P*_*nisA*_::*rbs*_*nisA*_::*sp*_Usp45_*-nucA-cbm3a-coh3-cwa*_M6_*-tlt2*	This Work

pAW329	Cm^r^, Amp^r^; pBS::pIL252::*t*_*trpA*_::*P*_*nisA*_::*rbs*_*nisA*_::*sp*_Usp45_*-nucA-cbm3a-coh3-tlt2*	This Work

pAW331	Cm^r^, Amp^r^; pBS::pIL252::*t*_*trpA*_::*P*_*nisA*_::*rbs*_*nisA*_::*sp*_Usp45_*-nucA-cbm3a-cwa*_M6_*-tlt2*	This Work

pAW332	Cm^r^, Amp^r^; pBS::pIL252::*t*_*trpA*_::*P*_*nisA*_::*rbs*_*nisA*_::*sp*_Usp45_*-nucA-cbm3a-tlt2*	This Work

pAW504	Cm^r^, Amp^r^; pBS::pIL252::*t*_*trpA*_::*P*_*nisA*_::*rbs*_*nisA*_::*sp*_Usp45_*-lacZα-cwa*_*M6*_*-tlt2*	This Work

pAW505	Cm^r^, Amp^r^; pBS::pIL252::*t*_*trpA*_::*P*_*nisA*_::*rbs*_*nisA*_::*sp*_Usp45_*-lacZα-tlt2*	This Work

pAW507	Cm^r^, Amp^r^; pBS::pIL252::*t*_*trpA*_::*P*_*nisA*_::*rbs*_*nisA*_::*sp*_Usp45_*-coh9-cwa*_M6_*-tlt2*	This Work

pAW508	Cm^r^, Amp^r^; pBS::pIL252::*t*_*trpA*_::*P*_*nisA*_::*rbs*_*nisA*_::*sp*_Usp45_*-coh9-tlt2*	This Work

pAW510	Cm^r^, Amp^r^; pBS::pIL252::*t*_*trpA*_::*P*_*nisA*_::*rbs*_*nisA*_::*sp*_Usp45_*-coh1-cwa*_*M6*_*-tlt2*	This Work

pAW511	Cm^r^, Amp^r^; pBS::pIL252::*t*_*trpA*_::*P*_*nisA*_::*rbs*_*nisA*_::*sp*_Usp45_*-coh1-tlt2*	This Work

pAW534	Cm^r^, Amp^r^; pBS::pIL252::*t*_*trpA*_::*P*_*nisA*_::*rbs*_*nisA*_::*sp*_Usp45_*-coh1-coh2-cwa*_M6_*-tlt2*	This Work

pAW535	Cm^r^, Amp^r^; pBS::pIL252::*t*_*trpA*_::*P*_*nisA*_::*rbs*_*nisA*_::*sp*_Usp45_*-coh1-coh2-tlt2 *	This Work

pAW528	Cm^r^, Amp^r^; pBS::pIL252::*t*_*trpA*_::*P*_*nisA*_::*rbs*_*nisA*_::*sp*_Usp45_*-cbm3a-coh3-cwa*_M6_*-tlt2*	This Work

pAW529	Cm^r^, Amp^r^; pBS::pIL252::*t*_*trpA*_::*P*_*nisA*_::*rbs*_*nisA*_::*sp*_Usp45_*-cbm3a-coh3-tlt2*	This Work

pAW531	Cm^r^, Amp^r^; pBS::pIL252::*t*_*trpA*_::*P*_*nisA*_::*rbs*_*nisA*_::*sp*_Usp45_*-cbm3a-cwa*_M6_*-tlt2*	This Work

pAW532	Cm^r^, Amp^r^; pBS::pIL252::*t*_*trpA*_::*P*_*nisA*_::*rbs*_*nisA*_::*sp*_Usp45_*-cbm3a-tlt2*	This Work

pETdock1	Kn^r^; pET28(b)::with cloned *dock1 *from *celS*	This Work

pETUdock1	Kn^r^; pET28(b)::*PT7*::*6xHis-uidA-dock1*	This Work

pETU	Kn^r^; pET28(b)::*PT7*::*6xHis-uidA*	This Work

^*a*^Vector pSCNIII was a gift provided by Jos Seegers (unpublished data)

pAW100 series of vectors are nisin-inducible and contain an intact *rbs*_*usp45*_. pAW300 series vectors are nisin-inducible and contain an intact *rbs*_*nisA*_. pAW500 series vectors are pAW300 variants lacking an N-terminal NucA fusion. *P59*, constitutive lactococcal promoter; *PT7*, inducible T7 promoter; *P*_*nisA*_, inducible *nisA *promoter; *rbs*_*usp45*_, Usp45 ribosome-binding site; *rbs*_*nisA*_, *nisA *ribosome-binding site; *sp*_Usp45_, signal sequence of Usp45; *nucA*, staphylococcal nuclease; *cwa*_M6_, anchor motif of M6 protein; *llt2*, transcriptional terminator of *rrnB *operon; *t*_*trpA*_, transcriptional terminator of *trpA*.

### Assembly of cassettes for scaffold protein expression and targeting

The *E. coli-L. lactis *shuttle vectors pVE5524 and pVE5523 were used as backbone plasmids for targeting fragments of the CipA scaffold protein to the cell surface or supernatant, respectively [36]. The various CipA_frags _were produced as fusions with the N-terminal signal peptide from the lactococcal Usp45 secreted protein (sp_Usp45_) and for targeting to the cell wall, as a fusion with the C-terminal anchor from the *Streptococcus pyogenes *M6 protein (cwa_M6_) (Fig. 1). Expression cassettes were designed to allow the optional fusion of CipA_frags _with an N-terminal nuclease reporter (NucA) used for detection of the fusion proteins in the extracellular milieu [35,38] (Fig. 1). The strong constitutive lactococcal promoter *P*59 [36] and the *P*_*nisA *_nisin-inducible promoter from the *nisA *gene of *L. lactis *[66] were tested for optimal expression of the recombinant scaffolds. Two ribosome-binding sites were also tested, that of the *usp45 *gene (*rbs*_*usp45*_) [36] and that of the *nisA *gene (*rbs*_*nisA*_) [66]. In order to facilitate the exchange of scaffold fragments in the expression cassette, *Asc*I-*Not*I restriction sites were engineered just downstream of *nucA *(Fig. 1). To achieve this, an 800-bp fragment containing the *nucA *gene was PCR-amplified from pVE5524 using primers *a *and *b *(Table 2), digested with *Sal*I-*Eco*RV and ligated into similarly digested pVE5524 and pVE5523, yielding pAW004 and pAW005. To facilitate detection of *E. coli *clones that harbor *cipA *fragments, a *lacZ*-α stuffer fragment was PCR-amplified from pUC19 using primers *c *and *d*, digested with *Asc*I-*Not*I, and subsequently ligated into similarly cut pAW004 and pAW005, yielding pAW004Z and pAW005Z, respectively. Since *L. lactis *HtrA NZ9000 is resistant to erythromycin, the *ery *marker of the pAW vectors was replaced with the *cat *gene from pSCNIII. The *cat *gene was PCR-amplified using primers *e *and *f*, digested with *Afl*II and *Hpa*I, and ligated into similarly digested pAW004Z and pAW005Z, yielding plasmids pAW004ZC and pAW005ZC, respectively. For inducible expression of the scaffolds, we replaced the *P59 *promoter with *P*_*nisA *_from pSIP502. The *P*_*nisA *_promoter was isolated using primers *o *and *p*, digested with *Apa*I-*Nru*I and ligated to similarly digested pAW004ZC and pAW005ZC, yielding pAW104 and pAW105, respectively.

**Table 2:** Primers used in this study.

Primer	Sequence (5'-3')
*a*	TAT**AGATCT**TCGATAGCCCGCCTAATGAGC
*b*	AT**GATATC**GCGGCCGCGGCGCGCCTCGAGATCGATTTG
*c*	TAGATATC**GGCGCGCC**ATTAGCTATGCGGCATCAGAGC
*d*	TAGCTAGC**GCGGCCGC**GCCCAATACGCAAACCGCCTC
*e*	GATCTAGC**CTTAAG**TTCAACAAACTCTAGCGCC
*f*	CGTAGATC**GTTAAC**CCTTCTTCAACTAACGGGG
*g*	TCGA**GGCGCGCC**CGGCCACAATGACAGTCGAGA
*h*	TCGA**GCGGCCGC**CGGTACGGAACTACCAAGAT
*i*	TA**GGCGCGCC**ATAAGTTGACACTTAAGATAGGCAG
*j*	TA**GCGGCCGC**AGTTACAAGTACTCCACCATTG
*k*	TCGA**GCGGCCGC**CGGTGTTGCATTGCCAACGT
*l*	TCGA**GGCGCGCC**CGGATGATCCGAATGCAATAAAG
*m*	TCGA**GCGGCCGC**TACTACACTGCCACCGG
*n*	TGA**GGCGCGCC**CGGCAAATACACCGGTATC
*o*	ATGC**GGGCCC**GACCTAGTCTTATAACTATACTG
*p*	ATGTAC**TCGCGA**TTTATTTTGTAGTTCCTTCGAACG
*q*	AGAACAG**TCATGA**AAAAAAAGATTATCTC
*r*	ATAT**CTCGAG**ATCGATTTGACCTGAATCA
*s*	AGTC**ACATGT**TCTTTCCTGCGTTATCCCCTG
*t*	ATGC**TCGCGA**AGATCTGGGATCAAAAAAAAGCCCGC
*u*	GCTT**GAATTC**TCTACTAAATTATACGGCGACGTCAATG
*v*	GCTT**GCGGCCGC**TTTAGTTCTTGTACGGCAATGTATC
*w*	ATGC**GCTAGC**ATGTTACGTCCTGTAGAAACC

Restriction enzyme cut sites are in bold.

### Cloning of *cipA *fragments from *C. thermocellum*

Five unique *cipA *fragments were PCR-amplified from *C. thermocellum *genomic DNA using primer pairs *g-h, i-j, g-k, l-m *and *n-m *(Table 2), ligated into pGEM-T (Promega) and sequenced to verify the integrity of the gene sequence. The resulting pGEM plasmids were digested with *Asc*I-*Not*I to release the *cipA *gene fragments and these were ligated into pAW004ZC and pAW005ZC. The *cipA *fragments were chosen on the basis of containing a single cohesin (coh1 or coh9), two cohesins of identical specificity (coh1-coh2), one cohesin and a cellulose-binding module (coh3-CBM3a) and only a cellulose-binding module (CBM3a) (Fig. 1). The resulting *sp*_Usp45_-*nucA-cipA*_frag_-*cwa*_M6 _cassettes were under control of the *P*_*59 *_promoter and contained *rbs*_*usp45*_. The same *cipA *fragments were cloned into pAW104 and pAW105 for inducible expression of the scaffold proteins.

For the inducible expression of the fusion proteins under the control of *P*_*nisA *_with an intact ribosome-binding site from the *nisA *gene (*rbs*_*nisA*_), *sp*_Usp45_-*nucA *was PCR-amplified from pAW004ZC using primers *q *and *r*, creating a *Bsp*HI cut site at the 5' end of the PCR product. The PCR product was digested with *Bsp*HI and *Xho*I and ligated to pSIP502 digested with *Nco*I-*Xho*I, effectively replacing the *gusA *gene with *sp*_Usp45_-*nucA*, retaining the first lysine of the signal peptide, and yielding pSIPSPNUC. For the insertion of an upstream transcriptional terminator and removal of *nucA*, a 1500-bp *Sap*I-*Xba*I fragment was temporarily removed from pAW104, and was ligated to similarly cut pUC19, yielding vector pUC104. To introduce the *E. coli *transcriptional terminator from the tryptophan synthase operon (*t*_*trpA*_) upstream of *P*_*nisA *_and to introduce a *Bgl*II cut site, a 200-bp fragment containing *t*_*trpA *_was PCR-amplified from pVE5524 using primers *s *and *t*, digested with *AflI*II-*Nru*I and ligated to similarly-cut pUC104, yielding pUC104mod. Plasmid pSIPSPNUC was digested with *Bgl*II-*Xho*I and ligated to similarly-digested pUC104mod, yielding vector pUC304. This was the base vector harboring the *t*_*trpA*_-*P*_*nisA*_-*rbs*_*nisA*_*-sp*_Usp45_*-nucA *cassette, which was digested with *Apa*I-*Asc*I and ligated into the pAW100 series of vectors. Inserting this cassette into *Apa*I-*Eco*RV digested pAW110 and pAW111, yielding pAW301 and pAW302, respectively, created controls lacking *cipA *fragments for expression of *nucA *alone. For deletion of the *nucA *reporter and construction of the pAW500 series, pUC304 was digested with *Sal*I-*Xho*I and self-ligated, yielding vector pUC504. The *t*_*trpA*_-*P*_*nisA*_-*rbs*_*nisA*_*-sp*_Usp45 _cassette was released via digestion with *Apa*I-*Asc*I, gel-purified, and ligated to similarly-cut pAW100 series vectors, yielding the pAW500 series of vectors. This cassette was also ligated into similarly cut pAW104 and pAW105 yielding base vectors containing the *lacZ-α *stuffer fragment. The final expression vectors for this study included the pAW300 series of vectors for inducible expression and targeting of NucA-fused scaffolds, and the pAW500 series of vectors for inducible expression and targeting of scaffolds lacking the N-terminal NucA reporter (Fig. 1).

### Expression and localization of CipA_frags _in *L. lactis*

*L. lactis *HtrANZ9000 was transformed with the pAW300 and pAW500 series of vectors for the controlled expression of scaffolds. It contains chromosomal copies of the *nisR *and *nisK *genes necessary for nisin-inducible expression of cassettes under control of the *nisA *promoter, and is deficient in a major extracellular housekeeping protease, which has been shown previously to be responsible for the proteolysis of exported recombinant proteins [37]. Growth curves were used to evaluate the potential of growth inhibition caused by the over-expressed CipA_frag _proteins. Growth curves were performed in 96 well plates and cells were induced with 10 ng nisin/mL at inoculation (t = 0 hrs), 4 hrs post-inoculation (t = 4 hrs) or were not induced. For the expression of CipA_frag _proteins in *L. lactis *HtrA NZ9000, overnight cultures were diluted 1/50 into fresh GM17 medium and were induced with 10 ng nisin/mL when an OD_600 _≈ 0.3 was reached (4 hrs). After 20 hrs growth, successful CipA_frag _secretion was evaluated using a nuclease assay consisting of spotting cells on TBD-agar and observing pink color formation [36]. For analysis of NucA-CipA_frag _proteins in various cellular locations, cell fractionation was performed as described previously [58], with the addition of lysostaphin (0.6 mg/mL) [67]. Aliquots of proteins were blotted on TBD-agar plates and formation of a pink color was analyzed after a 1-hr incubation at 37°C.

### Expression and purification of CipA_frag_-binding β-glucuronidase

The *E. coli *β-glucuronidase (UidA) was engineered to have a C-terminal dock1 module for binding onto CipA_frag _scaffolds, as well as an N-terminal 6 × His-tag for protein purification. The dock1 module of the *C. thermocellum celS *gene was amplified from *C. thermocellum *genomic DNA using primers *u *and *v *(Table 2). PCR products were digested with *Eco*RI-*Not*I and ligated to similarly-digested pET28(b), yielding pETdock1. The *uidA *gene lacking a stop codon was amplified using primers *w *and *x *and pSIP502 as template. The PCR product was digested with *Nhe*I-*Eco*RI and ligated to similarly-cut pET28(b) and pETdock1, yielding His-tagged UidA proteins with and without a dock1 module (pETUdock1 and pETU). His-tagged proteins were expressed in *E. coli *BL21(DE3). Cultures were induced at an OD_600 _of 0.5 with 1 mM IPTG and incubated for an additional 5 hrs at 37°C. Cells were harvested (1000 × g, 10 min, 4°C) and cell pellets were kept overnight at -80°C. Thawed cell pellets were suspended in 50 mM phosphate buffer, pH 7.5, containing 300 mM NaCl. Samples were subjected to sonication (15 sec pulse, 5 sec between pulses, 2 min total process time) and lysates were loaded on approximately 10 mL of Ni-NTA sepharose resin. The resin was washed with phosphate buffer (50 mM, pH 6.0) containing 300 mM NaCl and 20 mM imidazole and eluted using the same buffer containing 250 mM imidazole. Fifty μL of each elution fraction were added to 450 μL GUS buffer containing 50 mM sodium phosphate buffer (pH 7), 10 mM β-mercaptoethanol, 1 mM ethylenediaminetetraacetic acid and 0.1% (v/v) Triton X-100. Samples were heated for 1 min, after which *p*-nitrophenyl-β-D-glucuronide was added to a final concentration of 4 mg/mL [68]. The UidA-containing fractions were identified by the appearance of a yellow color. Proteins from the elution fractions showing UidA activity were visualized by SDS-PAGE on a 12% (w/v) gel to identify fractions containing the highest purity of enzyme. The specific activities of UidA-dock1 and UidA were determined by colorimetric assays in a thermostated UV-Vis spectrophotometer (Cary 50 WinUv) at 405 nm, using a 1 cm (L) cuvette, and the known molar extinction coefficient of *p*-nitrophenol being 18 000 M^-1 ^cm^-1^. Quantification of the proteins was done using a Bradford protein assay kit (Pierce) and BSA as a standard. Specific activities were used to evaluate the amount of enzyme bound to cells in the *in vivo *binding assay described below.

### Binding of β-glucuronidase to *L. lactis*

*L. lactis *HtrA NZ9000 cells harboring the pAW300 or pAW500 series of vectors, as well as the plasmid-free strain were grown overnight in GM17 medium. Cultures were diluted 1/50 in 5 mL of fresh media and grown for an additional 4 hrs (OD_600 _≈ 0.3) after which cells were induced with 10 ng nisin/mL for scaffold expression. After 20 hrs of growth, cells from 1-mL of culture were harvested (4,300 × g, 5 min, 4°C) washed once in phosphate buffer (50 mM, pH 6.0) containing 300 mM NaCl and suspended in 100 μL of purified UidA-dock1 or UidA at a concentration 100 μg/mL. To ensure that saturation of all cohesin sites was achieved, binding assay with 200 μg UidA-dock1/mL was tested for *L. lactis *harboring pAW328. Binding was carried out at 4°C for 10 hrs. Cells were then washed 6 times to eliminate residual enzyme activity and suspended in 100 μL of phosphate buffer (50 mM, pH 6.0) containing 300 mM NaCl for detection of β-glucuronidase activity. For quantification of bound UdiA-dock1, 50 μL of washed cells were analyzed for β-glucuronidase activity. Reactions were stopped with 250 μL of 1 M sodium carbonate once a yellow color appeared, and the duration of each assay was recorded. The specific activities of the purified UidA-dock1 and UidA were used to determine the amount of enzyme bound onto the *L lactis *cells. Using the calculated molecular weight of UidA-dock1 and the known amount of cells present in each sample, the average number of enzyme units bound per cell was estimated. Assuming a 1:1 cohesin to dockerin ratio, the number of enzymes present per cell also is a representation of the number of cohesins present on the cell surface. The calculated molecular weight of the scaffolds was used to estimate the net amount of recombinant protein anchored to cells in respective cultures. Experiments were repeated twice and true biological replicates (independent colonies and cultures) were performed in triplicate for all samples.

## Competing interests

The authors declare that they have no competing interests.

## Authors' contributions

VM defined the strategy described and supervised the project. AW designed and carried out all experiments. AW drafted the initial manuscript, VM helped draft the manuscript, and both AW and VM edited the manuscript. VM supervised the entire PhD project of AW. All authors read and approved the final manuscript.
